# Validation of a novel mask-based device for monitoring of comprehensive sleep parameters and sleep disordered breathing

**DOI:** 10.1007/s11325-025-03250-1

**Published:** 2025-01-20

**Authors:** Benjamin D. Fox, Murad Shihab, Abed Nassir, Dahlia Kushinsky, Ofer Barnea, Asher Tal

**Affiliations:** 1 Shamir Medical Center, Be’er Ya’akov, Israel; 2 Dormotech Medical, Dolev 4, Raanana, Israel; 3 Tel Aviv University, Chaim Levanon St 55, Tel Aviv-Yafo, Israel; 4 Soroka Medical Center, Yitzhack I. Rager Blvd. 151, Be’er Sheva, Israel; 5 Ben-Gurion University of the Negev, David Ben Gurion Blvd 1, Beer-Sheva, Israel

**Keywords:** Type II sleep test, Sleep disordered breathing, Apnea-hypopnea index, Polysomnography, Sleep parameters, Objective sleep measurements

## Abstract

**Purpose:**

This study aimed to validate the new DormoTech Vlab device’s performance, usability, and validity as a sleep test and physiological data recorder. The novel device has been designed for patient comfort, ease of use, and home-based assessment of sleep disordered breathing and other sleep-related measurements.

**Methods:**

Forty-seven adults (mean age = 52 years, 42% female, body mass index 29.4 kg/m^2^) underwent simultaneous testing with the DormoTech Vlab device and routine full polysomnography (PSG) using the Nox A1 system (K192469, Nox Medical). The sleep studies were manually and independently scored according to recommended guidelines. The primary outcome measure was the apnea-hypopnea index (AHI) and its corresponding conventional severity level (i.e., normal, mild, moderate, severe). Secondary endpoints included other standard PSG parameters.

**Results:**

The AHI was 21.7 ± 24.2 events/h (mean ± standard deviation) using the Vlab device versus 21.5 ± 23.9 events/h for gold standard PSG Nox A1 (*p* = 0.7). When AHI was grouped by severity, inter-test agreement was high (Cohen’s kappa = 0.97). Results between the two systems were largely similar in the secondary endpoints, with high correlation between the two systems, and statistically significant (*p* < 0.05) differences only in REM latency measurements. The Vlab device provides similar sleep study data to conventional gold standard PSG and clinically near-identical test interpretation in almost all cases.

**Conclusion:**

Based on these results, the Vlab device can be considered substantially equivalent to the reference Nox A1 system in terms of usability, efficacy, and validity.

**Clinical Trial Registration:**

Trial name: Evaluation of the Usability and Performance Assessment of the DormoTech VLAB Device as a Home Sleep Test Identification number: NCT06224972. Date of Registration: 2023-12-06.

**Supplementary Information:**

The online version contains supplementary material available at 10.1007/s11325-025-03250-1.

## Introduction

Sleep disorders are a prevalent concern in modern medicine, affecting a sizeable portion of the global population [1–3]. Chronic sleep disorders can lead to various health complications including cardiovascular diseases, metabolic disorders, cognitive impairments, and overall reduced quality of life [4].

Polysomnography (PSG) in the sleep laboratory with an attending technician is considered the gold standard for sleep disorder diagnosis. In-lab PSG studies require patients to stay overnight in a specialized sleep laboratory, which is often inconvenient for patients and may not represent their typical sleep environment nor accurately reflect their typical sleep architecture or circadian rhythm [5]. In addition, in-lab PSG sleep studies are often expensive with high costs for both clinicians and patients, and cumbersome, requiring patients to be attached to extensive wiring which may increase anxiety or discomfort and cause inaccurate results during the sleep study.

Accurate home sleep studies are therefore becoming a more desirable option, especially for the diagnosis of common sleep disorders such as restless leg syndrome (RLS) or obstructive sleep apnea (OSA) [6–8]. However, many sleep study devices designed for at-home-use include only a subset of standard PSG physiological measurements, such as pulse oximetry, snoring volume, and heart rate [9, 10], potentially leading to inconclusive, incomplete, or false results [10]. Consequently, there is a growing need for the development of new, home-use devices that can accurately monitor the full extent of PSG-related signals that include not only oxygen saturation, heart rate, and snoring volume but also EEG, chin EMG, EOG, respiratory effort, airflow, and others. Moreover, several of these devices are not user friendly, with complicated setup instructions that often require patients to arrive at the sleep lab in the evening to receive assistance from a sleep technician in the setup and wearing of the device. Other devices may be bulky or uncomfortable, which can limit their practicality or usefulness in at-home unsupervised settings. Additionally, due to high costs and limited resources, current standard practice typically involves testing during a single night which may negatively impact the validity of results or render them inconclusive [11, 12].

Given these limitations, there is a growing need for a more user-friendly, accurate, and cost-effective method to diagnose sleep disorders in the patient’s home. This method should allow patients to attach the device easily and independently at home, and permit remote monitoring of data. Such a home device could potentially be retained by the patient and reused for improving diagnoses or monitoring the effects of treatment on sleep signals.

**Fig. 1:**
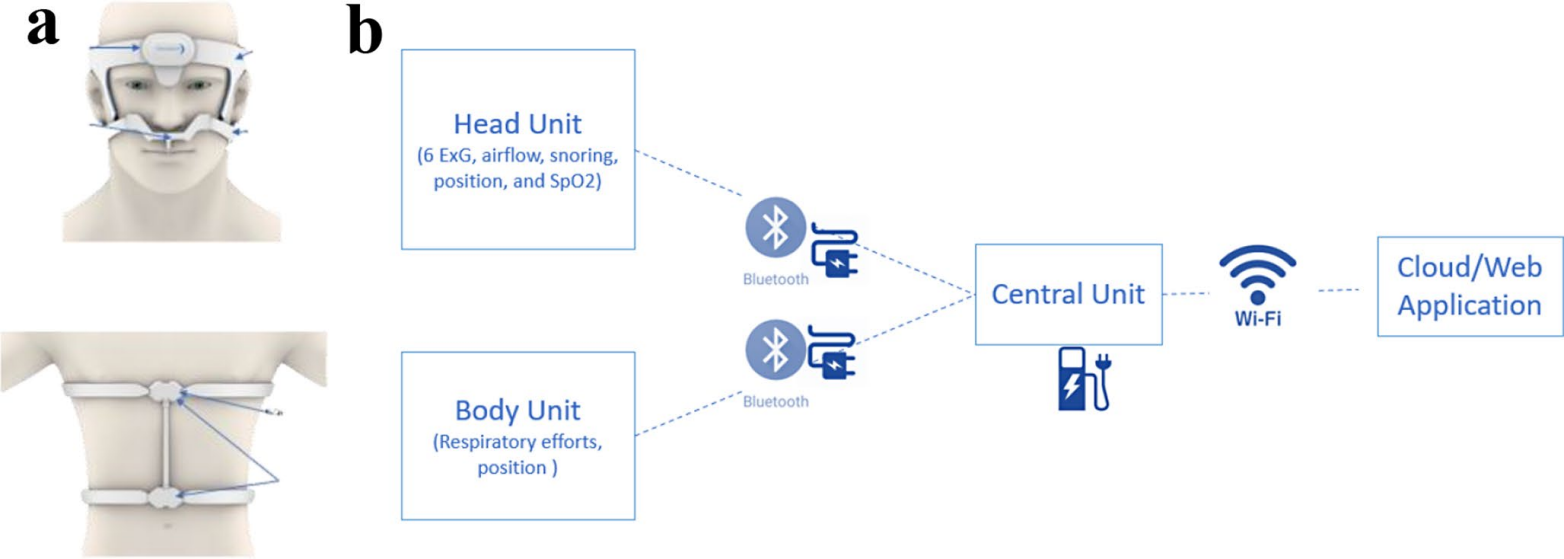
Vlab device and signal pathway from measurement to cloud. **a** Illustration of Vlab device and placement on head and body for full type 2 sleep study recording. **b** Physiological signals are collected from the head and body unit and passed to the central unit via Bluetooth. Signals are uploaded to the cloud or web application via the local WIFI signal

The FDA-cleared DormoTech Vlab device was developed to fit these key criteria, with the aim of providing a home-based solution for comprehensive sleep monitoring at the same level of data quality and validity as formal in-lab PSG measurements. The DormoTech Vlab device is a wearable, wireless physiological data recorder designed for easy patient self-application, allowing for fast, comfortable, and flexible installation and data collection while retaining all PSG signals (Fig. 1A). Vlab monitors and records oxygen saturation, heart rate, EEG, EOG, EMG, total airflow, snore volume, respiratory effort, and positioning of both body and head. Notably, in this study the DormoTech Vlab focuses solely on data recording while data management and analyses are conducted by external suppliers. The device contains an integrated head unit which is installed on the patient’s head and a body unit worn around the torso, all of which communicate wirelessly with the central unit. The central data collection unit transfers data from head and body units continuously in real time via local WIFI (Fig. 1B).

The Vlab device differs from other previously available PSG devices both for at-home and in-lab use. These differences are primarily seen in its simple design which easily allows patients to self-apply the device, its comprehensive set of PSG measurements, and its relatively low cost, which gives physicians and patients greater access to high-quality and comprehensive sleep studies. While other FDA-approved at-home devices such as the Alice PDx (Philips) may cause frustration or confusion during self-application due to the need to self-attach several separate leads on different body parts, the Vlab device is entirely wireless and can be self-applied in less than one minute. Other readily used devices, such as the Nox A1s, can be used both in the sleep clinic and at home, but involve the attachment of several separate units to the body with multiple wires, which may cause anxiety or complications before or during sleep. The design of the Vlab device reduces these potential complications due to its simple structure, which houses the leads for all needed signals and does not require patients to apply several separate units or have exposed wires throughout the night.

In this study, we aimed to assess the agreement between the newly developed DormoTech Vlab and gold-standard PSG systems by conducting simultaneous physiological measurements using both the Vlab and a gold-standard PSG system (Nox A1) in a sleep laboratory setting. This simultaneous recording scheme allowed for direct comparison between the two devices to assess the level of agreement.

## Materials and methods

### Study design and overview

The primary objective of this study was to evaluate the validity and level of agreement between the new DormoTech Vlab device and gold-standard Nox A1 PSG. The study was conducted between June 2023 and August 2023 and was a comparative, observational clinical trial. Participants were recruited during attendance at the Shamir Medical Center Sleep Laboratory (Be’er Yaakov, Israel) or Millenium Sleep Clinic (Be’er Sheva, Israel) where PSG is routinely performed using the Nox A1 system (Nox Medical, Reykjavik, Iceland, K192469). All participants had been referred for PSG by their physician and were not selected in advance by the investigators. All participants gave informed consent prior to their inclusion in the study. Inclusion criteria included participant willingness and ability to comply with protocol requirements, giving informed consent, and an age restriction of 22 years or older. Participants were excluded if they were not willing to sign informed consent, had implanted electronic devices or used exterior electronic devices during the procedure, had known allergies to device materials, had skin irritation or open wounds at the device placement site, or were pregnant. All procedures were approved by the IEC committee on ethics of Shamir Medical Center on April 24, 2023, study number 0067-23-ASF.

Participants consenting to the study were required to wear the DormoTech Vlab device in parallel with the Nox system and complete questionnaires both pre- and post-study. Attending laboratory technicians applied both the Nox and Vlab devices on the patients. The device was worn throughout the night and data from both devices were collected for analysis. Participant sample size was estimated following EP09-A3 and FDA guidance. A total of 47 participants were recruited across two sites. Following descriptive analyses, 2 participants were excluded due to technical issues (i.e. device not being fully charged) during the study and 2 participants redacted their consent during the sleep study. Of the remaining participants, 2 patients were defined in advance by the researchers as pre-test cases and were therefore not part of the clinical data analysis.

### Recording process and data extraction

Conventional PSG recordings were performed using the Nox A1 device and Noxturnal Software System (version V.6.3). Vlab recordings were performed using the Vlab device. Recordings from both devices were saved on the laboratory’s local server and was accessible only by the laboratory technician. Separate EDF files from both devices were exported for scoring in the Noxturnal Software System. Signals recorded from the Vlab device were not modified or filtered before being exported to EDF formatting for accurate analysis using Noxturnal software.

Channels recorded by the Vlab device included electroencephalogram (EEG), electrocardiogram (ECG), electrooculogram (EOG), electromyogram (EMG), airflow, respiratory sound/snore, oxygen saturation, heart rate, respiratory effort, and body position. The gold-standard device, Nox A1, included channels recording EEG, EOG, and EMG, pressure, respiratory effort, airflow, respiratory sound/snore, position, and ambient light.

### Sleep study scoring

Sleep staging scoring for both recordings (Vlab and Nox) was performed using Noxturnal Software System (version V.6.3), allowing for manual scoring. In this study, all data was manually scored using the American Academy of Sleep Medicine guidelines, and respiratory events were scored according to AASM scoring rules. An apnea was scored if airflow was absent for 10s and a hypopnea if airflow dropped by ≥ 30% of pre-event baseline in association an arousal or an oxygen desaturation of 3% or 4%, according to recording location. Results from these two rules were compared and found to be similar, so were combined in population analyses.

Two trained PSG scorers, one for each site, performed the scoring. The same scorer scored both Vlab and Nox recordings for each participant at their site. Sleep scoring was performed blind to participant identification, as EDF files had no identifying information other than the participant’s study ID. Data from the two simultaneously recorded tests were not scored sequentially or on the same scoring session.

### Collected measurements

Descriptive statistics, including age, BMI, and sex were calculated to characterize all participants. Categorical variables (e.g., active smokers, sex) were presented as counts or frequency distributions. Continuous measurements were summarized as mean (± standard deviation).

The primary endpoint measurement was the apnea-hypopnea index (AHI) and severity level, calculated as the average frequency of apnea and hypopnea events, measured per hour of sleep. AHI severity was then categorized based on accepted severity levels (normal AHI < 5, mild 5–14, moderate 15–29, severe > 30).

The secondary endpoint measurements included other PSG parameters including total sleep time (TST, in minutes), total recording time (TRT, in minutes), sleep efficiency (%, TST/TRT × 100), sleep stages (Wake, N1, N2, N3, REM, in % of total recording time), sleep latency (in minutes), wake after sleep onset (in minutes), REM latency (in minutes), ODI (oxygen desaturation index), total snore (%), and body position (supine position, left, right, up, as % of TST). In patients who did not have REM sleep, REM latency was set to 0.

Device usability was assessed using pre- and post-study questionnaires based on a 5-point Likert scale. Participants were instructed to evaluate only the Vlab device, independent of the Nox A1 device. If a participant felt they could not differentiate between the devices when answering the post-night test questionnaire, this observation would be noted in our results. See Supplementary B for pre- and post-study questionnaires.

The number of Adverse Events (AE) related to device use was measured as a readout of device safety. While PSG is regarded as relatively safe, systematic evaluation of adverse events have suggested that AEs may occur including those cardiac in nature (mostly involving acute chest pain), falls, neurologic, pulmonary, or psychiatric in nature [13, 14].

### Acceptance criteria

Agreement between continuous parameters from the two different devices was analysed primarily using the Bland-Altman method. For primary endpoint analysis, the study protocol defined a priori < 15% deviation between AHI calculated from the different devices as being functionally equivalent in continuous AHI values. This functional equivalence threshold was chosen as diagnostic severity thresholds are relatively broad (normal AHI < 5, mild 5–14, moderate 15–29, severe > 30) and therefore a < 15% deviation in AHI values would not be likely to result in a classification difference. Moreover, previous clinical studies [15] have defined minimal clinically important differences in AHI > 10–15 events per hour, which would not be reached with our stringent threshold of < 15%.

In addition, we performed statistical t-tests and a priori defined confirmation of the null hypothesis (i.e. *p* > 0.05 in comparative tests) as an indication of equivalence between the two devices. For correlation analyses, we defined a priori a strong positive correlation of Pearson’s correlation coefficient as > 0.80. Device safety was assessed based on the frequency of adverse events (AEs). The acceptance criteria were decided by the physician according to the severity of the event.

Device usability was assessed via questionnaires (see Supplementary B) using a Likert type 5-point scale. We defined a priori that a score of 3 (neutral) or higher should be achieved for at least 70% of the questions in both questionnaires.

### Statistical analysis techniques

#### Qualitative measures

For qualitative measurements, the overall agreement between the DormoTech Vlab and the Nox device was calculated using binary determinations. Specifically, this involved comparing the categorization of sleep apnea severity (normal, mild, moderate, or severe) based on AHI between the two devices for each individual patient. The percentage of cases where both devices agreed on the severity was calculated.

#### Quantitative measures

All analyses adhered to FDA Guidance E6 GCP, E9, and ISO 14155 [16, 17].

Advanced statistical techniques, including Student’s t-test, Bland-Altman plots, Pearson’s correlation, and Passing-Bablok linear regression models were employed to compare the performance of the DormoTech Vlab and Nox A1 PSG devices. To provide a holistic view of the accuracy and validity of the measurements, the Root Mean Square Error (RMSE) was computed. A low RMSE indicates high agreement between the two devices.

Bland-Altman plots, also known as mean-difference plots, were used to analyse the agreement between the two measurements of the same continuous parameter. Specifically, for each metric we calculated the participant-specific difference and average value between the two recording methods. Values close to zero on the Bland-Altman plot suggest good agreement between the parameters. The limits of agreement (LoA) between the DormoTech Vlab and Nox A1 devices was defined as 2 standard deviations from the mean of the difference in score between the two systems. The upper and lower limits of agreement therefore represent the range where 95% of the measurement differences lie, with a narrower range indicating better method agreement. For example, the theoretical AHI range is 0-150 events per hour (theoretical maximum at 2.5 events per minute as each event must be at least 10s and occur during sleep). Recently, extremely severe cases of AHI were reported at 130–140 [18, 19]. As the calculated 95% LOA for AHI is 7 events per hour, and the possible range is 0-150 events per hour, then the 95% LOA is only 4.7% of the clinical theoretical range, indicating high agreement between the two devices.

The Cohen’s kappa coefficient was calculated to assess the agreement between the two raters, with values close to 1 indicating excellent agreement.

Passing-Bablok regression was calculated for measurements from the Vlab and Nox systems to assess the agreement between the two recording devices. The resulting intercept and slope are crucial parameters, where an intercept close to zero coupled with a slope nearing one signifies very high agreement and little bias between the two devices.

The Pearson’s correlation coefficient quantifies the linear relationship between the two devices, where a value of one indicates perfect relationship between the two signals.

## Results

The study enrolled 47 patients across two different sites: 33 patients at Site A (Shamir Medical Center, Be’er Ya’akov, Israel) and 14 patients at Site B (Millennium Sleep Clinic, Be’er Sheva, Israel). Participants were selected based on predefined inclusion and exclusion criteria. Table 1 summarizes key demographic metrics of the participants. Forty-five of forty-seven participants (95.7%) completed a full night of data collection with both devices. The participants were an average age of 52.2 years (SD = 12.8), while 7 participants (16%) were below the age of 40. Participants were primarily male (58% of all participants) and had an average body mass index (BMI) of 29.4 kg/m^2^ (SD = 5.3). Of all participants, 13% (6 participants) reported having diabetes, 4.3% (2 participants) reported having OSA, 15.0% (7 participants) reported having hypertension, and 21.3% (10 participants) reported other comorbidities (including obesity, asthma, hyperthyroidism, and others; see Supplementary A for details).

**Table 1:** Demographic and Baseline Analysis. BMI = body mass index, SD = standard deviation

**Description of the enrolled participants* (n*** = ***47)***		
Participants that expressed interest and enrolled the study (count, % of participants enrolled)	47	100%
Participants that completed the study (count, % of participants enrolled)	45	95.7%
Dropouts (count, % of participants enrolled)	2	4.3%
**Description of the participants completed the study* (n*** = ***45)***		
Age (mean, SD)	52.2	12.8
Sex, female (count, %)	19	42%
Age below 40 years of age (count, %)	7	15.5%
BMI kg/m^2^ (mean, SD)	29.4	5.3
Active smokers (count, %)	9	20%
Enrolled participants that contributed data in the final analysis set (count, %)	41	91.1%
Reported adverse events (count, %)	0	0%
**Cross-tabulation of sex by age group* (n*** = ***45)***		
	> 40 years	< 40 years
Female	16	3
Male	22	4

**Fig. 2:**
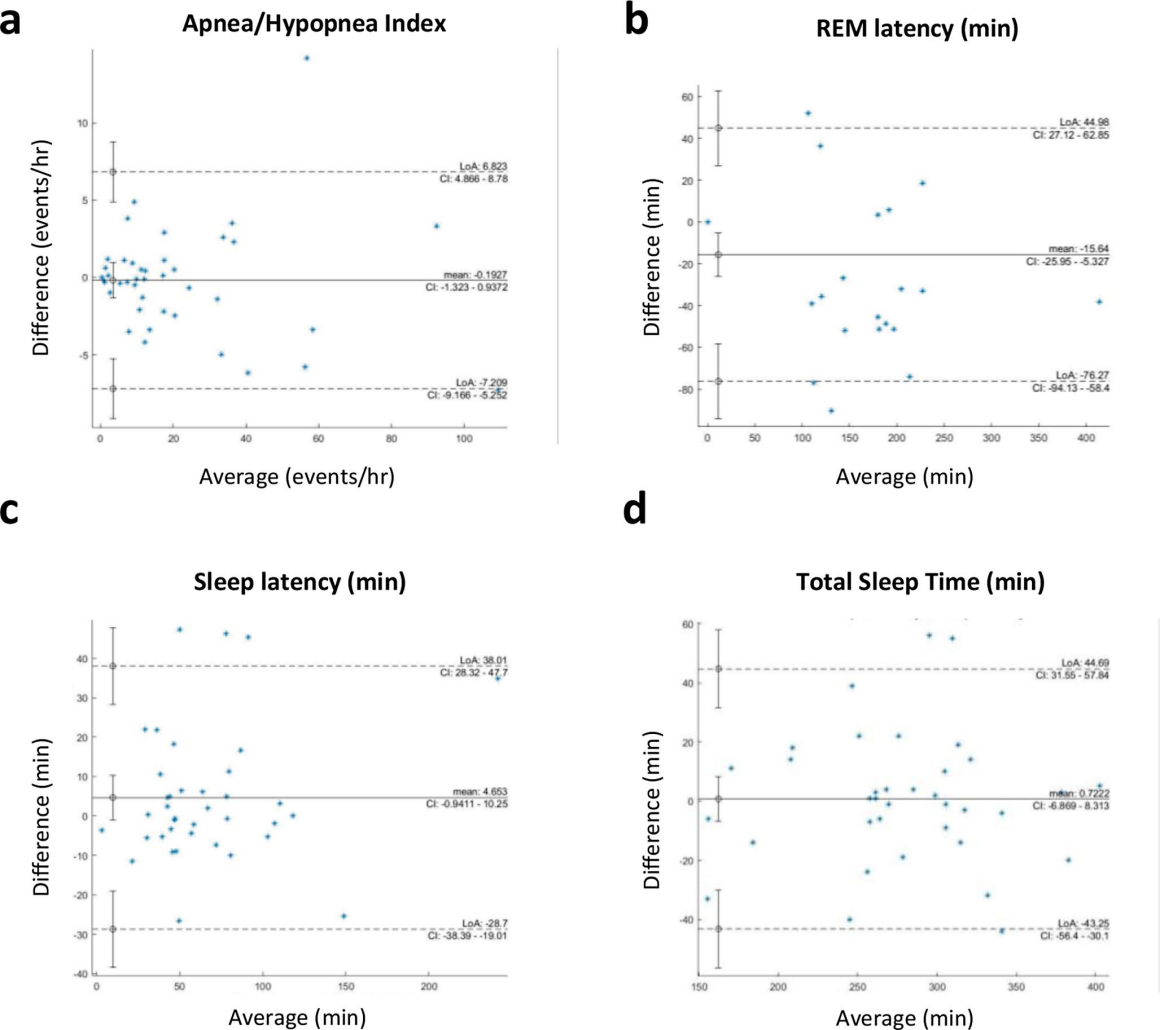
Bland-Altman plots demonstrate agreement between Nox and Vlab systems. **a-b**. Bland-Altman plots were calculated to compare the difference in scores between Nox and Vlab devices. LoA- limits of agreement, CI – confidence interval **a** AHI comparison **b** REM latency comparison **c** Sleep latency **d** Total sleep time

We compared the results from the simultaneously recorded Nox and Vlab methods (Table 2). The primary endpoint of the clinical study, absolute AHI value and severity level, was calculated for both devices separately. Overall, AHI values were not significantly different between Vlab and Nox device recordings (Nox: 21.5 ± 23.9, Vlab: 21.7 ± 24.2, mean ± SD, *p* = 0.7). To further compare the similarity between Vlab and Nox recordings, we calculated the root mean squared error (RMSE). This measurement also indicated high similarity between AHI scores as derived from Vlab and Nox devices (Table 2). Bland-Altman difference, LoA, and plots of AHI further confirm a high agreement between the two recording devices (Table 2; Fig. 2A). Additional analyses including, RMSE and Bland-Altman differences of secondary endpoints, for example REM latency, sleep latency, and total sleep time, showed high agreement between the two devices (Table 2; Fig. 2B-D).

**Table 2:** Descriptive statistics of sleep parameters extracted from the Nox and Vlab Devices. AHI = apnea-hypopnea index, ODI = oxygen desaturation index. REM = rapid eye movement, SD = standard deviation, RMSE = root mean squared error, LoA = limits of agreement

Parameter	Nox Mean ± SD	Vlab Mean ± SD	*P* value	RMSE	Bland-Altman Difference (LoA)
AHI (events/h)	21.5 ± 23.9	21.7 ± 24.2	0.7	3.5	-0.2 (6.8, -7.2)
ODI (events/h)	21.0 ± 26.7	21.3 ± 26.5	0.4	2.5	-0.3 (4.5, -5.2)
Snore (%)	32.9 ± 19.7	34.0 ± 19.6	0.1	4.6	1.1 (10.0, -7.8)
Sleep Latency (min)	68.3 ± 44.0	63.7 ± 41.0	0.1	17.4	4.7 (38.0, -28.7)
REM Latency (min)	83.9 ± 96.9	99.6 ± 111.4	0.004	34.3	-15.6 (45.0, -76.3)
Wake after Sleep Onset (min)	43.5 ± 31.4	47.8 ± 31.2	0.2	18.7	-4.3 (31.8, -40.4)
REM (%)	6.6 ± 8.3	6.1 ± 6.9	0.5	3.9	0.5 (8.1, -7.2)
N1 (%)	37.9 ± 22.2	37.6 ± 20.4	0.8	6.5	0.3 (13.2, -12.6)
N2 (%)	46.9 ± 15.2	49.7 ± 14.7	0.06	8.3	-2.5 (13.0, -18.0)
N3 (%)	7.9 ± 8.8	6.7 ± 7.6	0.07	3.2	1.0 (7.5, -5.5)
Wake (%)	28.5 ± 13.6	28.3 ± 13.4	0.8	4.4	0.2 (8.9, -8.5)
Total Sleep Time (min)	278.8 ± 60.3	278.1 ± 60.2	0.8	22.1	0.7 (44.7, -43.3)
Sleep Efficiency (%)	71.5 ± 13.6	71.5 ± 13.4	1.0	4.4	-0.03 (8.7, -8.7)
Position (Up) (%)	2.6 ± 3.4	2.6 ± 2.9	0.96	1.4	0.013 (2.9, -2.8)
Position (Supine) (%)	43.1 ± 26.6	42.1 ± 25.5	0.1	4.2	1.0 (9.0, -7.0)
Position (Left) (%)	21.1 ± 16.3	20.7 ± 15.7	0.6	4.1	0.4 (8.4, -7.7)
Position (Right) (%)	31.8 ± 25.2	32.2 ± 26.2	0.5	3.7	-0.4 (6.8, -7.6)

We next calculated severity levels based on absolute AHI values for each device independently. These levels were largely similar between both devices (Nox: 7, 16, 7, and 11 participants; Vlab: 7, 15, 8, and 11 in Normal, Mild, Moderate, Severe groups, respectively, Table 3). Only results from one participant (1/41 participants, 2.4% of all fully analysed studies) were categorized differently between the two recording method, graded as mild (AHI = 11.8) in the Nox system but moderate (AHI = 15.2) by Vlab. To assess the validity of the scorers, we calculated the Cohen’s Kappa coefficient and found that the validity of the scoring process was very high, at 0.97.

**Table 3:** Confusion matrix of AHI Severity according to NOX and Vlab devices. AHI = apnea-hypopnea index

		Vlab				
		Normal	Mild	Moderate	Severe	Total (Nox)
NOX	Normal	7	0	0	0	7
	Mild	0	15	1	0	16
	Moderate	0	0	7	0	7
	Severe	0	0	0	11	11
	Total (Vlab)	7	15	8	11	41

We also measured differences in the secondary endpoints between Nox and Vlab measurements, including ODI, snore, sleep latency, REM latency, total sleep time, sleep stages, sleep efficiency, and position. Of all measured secondary endpoints, we found one measurement, REM latency, was significantly different between the two systems (Nox: 83.9 ± 97.0, Vlab: 99.6 ± 111.4, mean ± SD, *p* = 0.004), while all other measurements were not significantly different (*p* > 0.05, Table 2). Additionally, no systematic differences were found in primary or secondary endpoints when comparing results from the two separate testing sites. Additional statistical analyses, including Bland-Altman difference and RMSE calculations confirmed the similarity in results from the two methods, with the largest differences being in REM latency measurements (Table 2; Fig. 2).

**Fig. 3:**
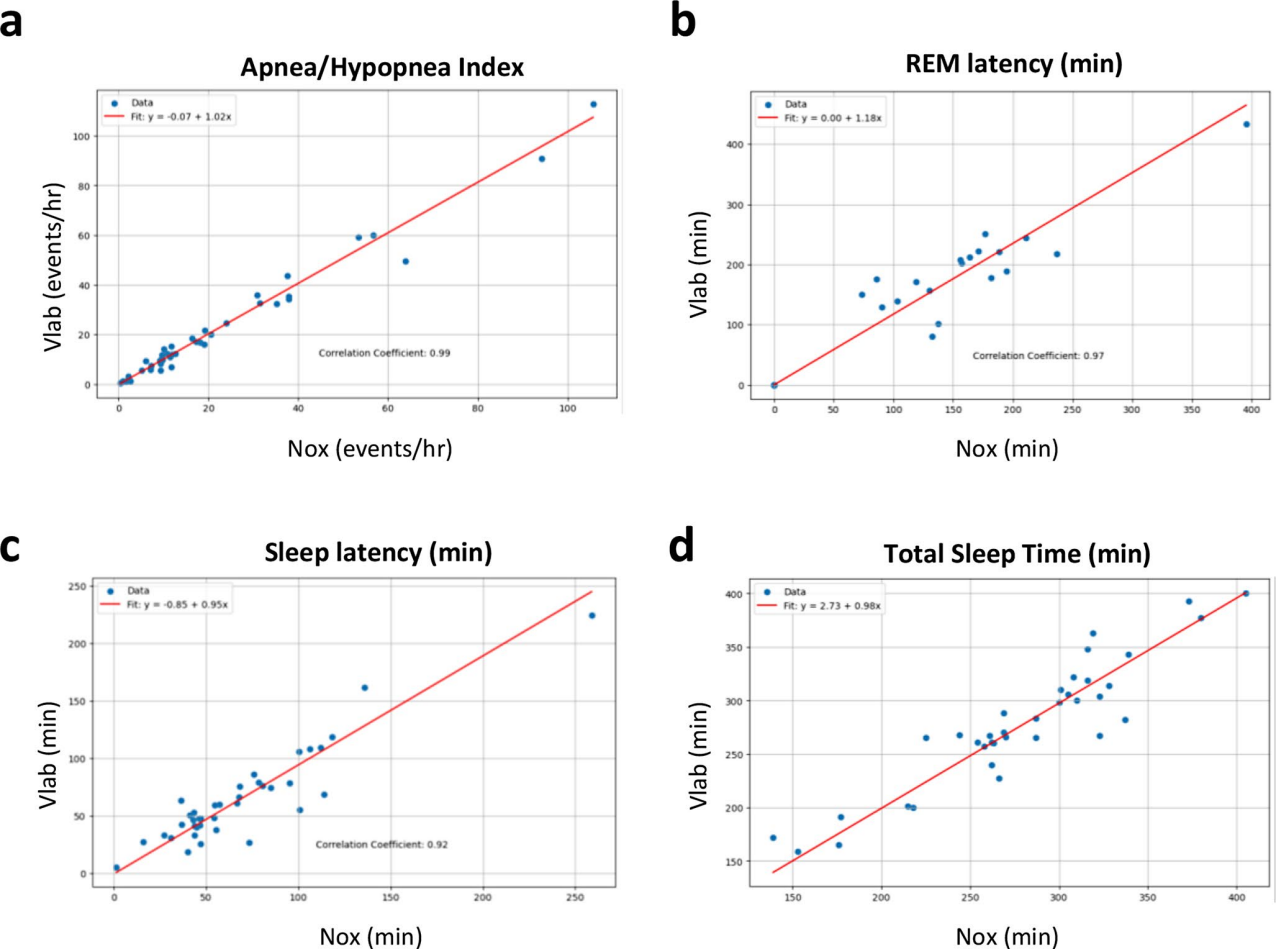
Passing-Bablok Regression demonstrates agreement between Nox and Vlab systems. Regression and Pearson’s correlation were calculated for individual participants between NOX and Vlab recordings. **a** AHI – apnea-hypopnea index. **b** REM latency **c** Sleep latency **d** Total sleep time

To further compare measured parameters between the Nox and Vlab devices, we calculated Passing-Bablok regression and Pearson’s correlation between results from the two recording methods. Passing-Bablok regression between the Nox and Vlab methods for the primary endpoint AHI was 1.02 (Fig. 3A; Table 4), and the correlation was very high at 0.99. Moreover, all secondary endpoint measurements had slopes > 0.84, indicating a high similarity between the Nox and Vlab methods (Fig. 3B-D; Table 4). Parameters such as ODI, REM, and Position (Left) had intercepts nearing zero, indicating a negligible bias between the Nox and Vlab methods for these metrics, while other secondary measurements had larger intercepts indicating a slight bias in recording methods. Pearson’s correlation analyses demonstrated a strong concordance between all parameters, ranging from 0.83 to 1.00, surpassing our a priori definition of high correlation, and therefore all parameters were in agreement between the two devices.

**Table 4:** Summary table of passing-Bablok regression of compared parameters. AHI = apnea-hypopnea index, ODI = oxygen destauration index, REM = rapid eye movement

Parameter	Intercept	Slope	Correlation Coefficient	Agreement
AHI (events/h)	-0.07	1.02	0.99	Agreement
ODI (events/h)	0.3	1.0	1.00	Agreement
Snore (%)	1.1	1.01	0.97	Agreement
Sleep Latency (Min)	-0.85	0.95	0.92	Agreement
REM Latency (Min)	0.0	1.18	0.97	Agreement
Wake after Sleep Onset (Min)	3.16	1.01	0.83	Agreement
REM (%)	0.0	0.88	0.89	Agreement
N1 (%)	2.41	0.92	0.96	Agreement
N2 (%)	3.50	0.99	0.86	Agreement
N3 (%)	0.0	0.85	0.94	Agreement
Wake (%)	-1.45	1.07	0.95	Agreement
Total Sleep Time (Min)	2.73	0.98	0.93	Agreement
Sleep Efficiency (%)	-5.13	1.06	0.95	Agreement
Position (Up) (%)	0.32	0.88	0.91	Agreement
Position (Supine) (%)	0.95	0.96	0.99	Agreement
Position (Left) (%)	0.0	0.97	0.97	Agreement
Position (Right) (%)	-0.11	1.04	0.99	Agreement

**Fig. 4:**
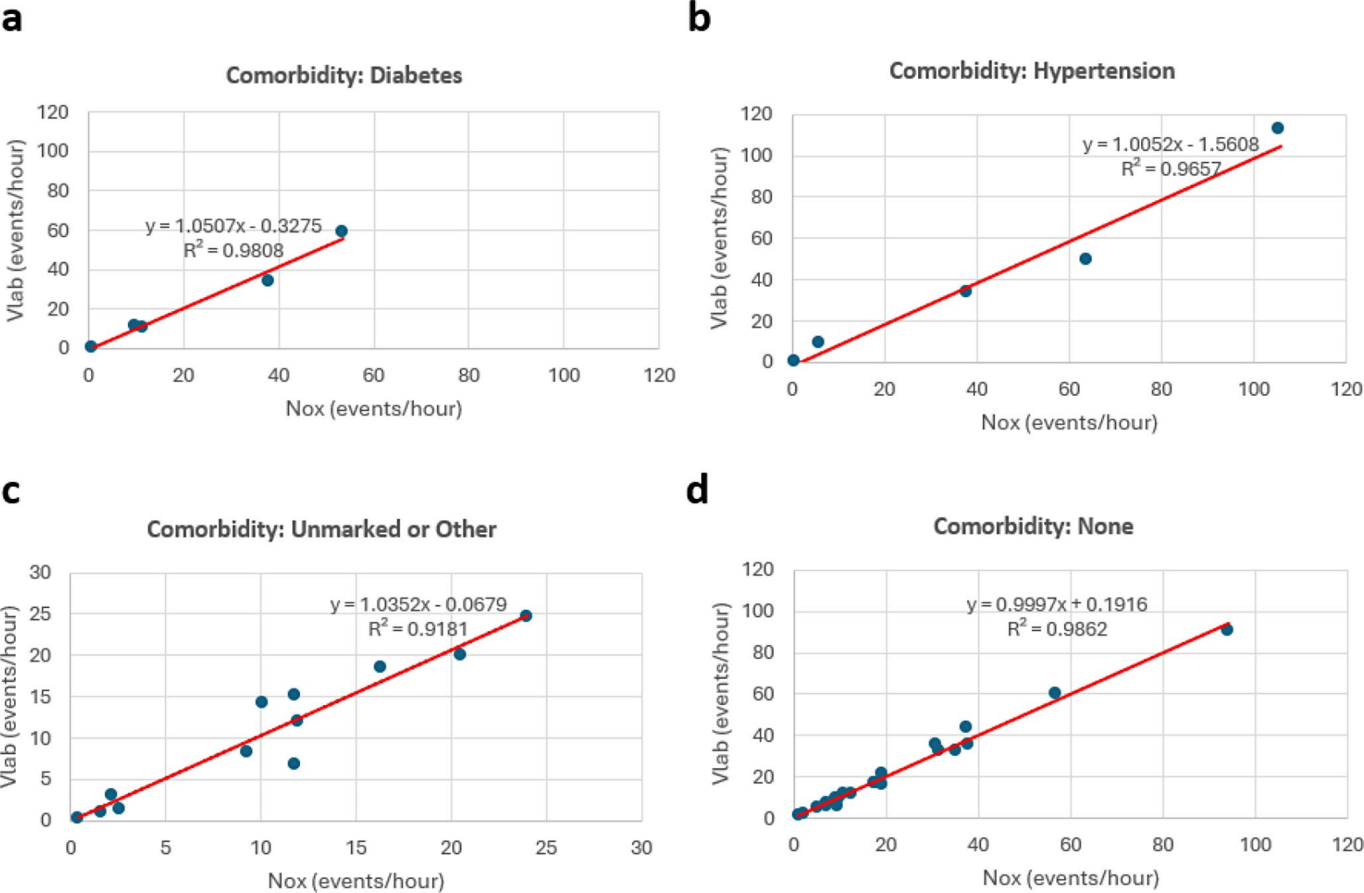
Passing-Bablok Regression analysis of AHI demonstrates agreement between Nox and Vlab systems across comorbidities. Regression and Pearson’s correlation were calculated between NOX and Vlab recordings according to participant comorbidities. **a** Patients self-reporting diabetes. **b** hypertension **c** unmarked or other comorbidity **d** reported no comorbidities

While the number of participants included in the data presented in this clinical study was relatively small, several participants reported comorbidities such as diabetes, hypertension, or other. To determine if these comorbidities had an impact on the performance of the DormoTech Vlab device or on the overall primary endpoint, AHI, we performed subgroup analyses. Participants were subcategorized into cohorts according to their reported comorbidities, including diabetes, hypertension, other/unreported, or none (Fig. 4). This analysis included small numbers of participants per group, but revealed no significant differences between the Nox and Vlab-derived AHI values in any of the analyzed subcategories (*p* > 0.05 in all groups, Fig. 4). Moreover, we performed correlation and Passing-Bablok Regression analyses to further probe if there were systematic differences in device reliability across comorbidity groups. This analysis revealed high correlations (ranging from 0.91 to 0.99), further illustrating the high similarity in scores between the two devices across reported comorbidities. Lastly, this analysis revealed larger average AHI values in patients with hypertension as compared to patients reporting no comorbidities (mean Vlab hypertension = 41.5; mean Vlab none = 22.6), however, these differences were not statistically significant (*p* = 0.17) likely due to low number of patients per group.

Finally, to assess device usability, we quantified participants’ experience via completion of usability questionnaires before and after the overnight sleep study, focused on patients’ experience with the Vlab device. The first, a 16-question survey using a 5-point scale ranging from 1 (Disagree strongly) to 5 (Strongly agree), was administered before the study. The second, an 18-question survey using a similar 5-point Likert scale, was given after the study. The average results from both surveys are summarized in Table 5 (see Supplementary B for full questionnaire). Overall, feedback questionnaires revealed that participants had a positive experience using the Vlab device. Most questions received scores that were biased toward “Agree” or “Strongly Agree” (of all responses, morning = 4.29 ± 0.40, evening = 4.42 ± 0.27, mean ± SD) indicating overall satisfaction with the device’s usability, comfort, and setup both before and after the sleep study (Table 5). The results surpass the study’s benchmark of 3 (Neutral) or higher for at least 70% of all survey questions.

**Table 5:** Usability Analysis of Vlab device based on subjective questionnaires. Shortened summary of each question is written below the question number, full questionnaires can be found in supplementary B

Question	Morning (after sleep)				Night (before sleep)				
	Min	Max	Average	% > 3		Min	Max	Average	% > 3
Question 1 (Head unit comfort)	1	5	3.8	0.84		2	5	4.1	0.98
Question 2 (Head unit stability)	2	5	4.3	0.98		3	5	4.5	1.00
Question 3 (Head unit no discomfort)	1	5	3.7	0.82		1	5	3.9	0.89
Question 4 (Head unit adaptation)	1	5	3.9	0.86		2	5	4.2	0.96
Question 5 (Nasal congestion)	1	5	4.3	0.93		1	5	4.6	0.93
Question 6 (Body unit comfort)	2	5	4.6	0.98		3	5	4.8	1.00
Question 7 (Body unit stability)	2	5	4.7	0.98		3	5	4.8	1.00
Question 8 (Body unit no discomfort)	2	5	4.7	0.99		1	5	4.5	0.93
Question 9 (Body unit adaptation)	2	5	4.6	0.99		1	5	4.7	0.98
Question 10 (Instructions were clear)	3	5	4.7	1.00		3	5	4.7	1.00
Question 11 (Instructions were easy to follow)	1	5	4.1	0.94		2	5	4.2	0.97
Question 12 (User manual was clear)	3	5	4.5	1.00		3	5	4.6	1.00
Question 13 (Operation was easy and clear)	3	5	4.6	1.00		2	5	4.4	0.97
Question 14 (Connectivity was easy and clear)	3	5	4.5	1.00		3	5	4.5	1.00
Question 15 (Useability without assistance)	1	5	4.0	0.92		2	5	4.0	0.88
Question 16 (Night: Overall rating of device Morning: Device remained in place)	2	5	4.7	0.98		3	5	4.3	1.00
Question 17 (Device didn’t interrupt sleep)	1	5	3.4	0.71					
Question 18 (Overall rating of device)	1	5	4.1	0.91					

## Discussion

We performed a meticulous comparative analysis of sleep signals and parameters captured simultaneously by two devices: the novel DormoTech Vlab and Nox A1 (a gold standard PSG device). Overall, the results of this validation study in 47 patients show that the Vlab device has high agreement with gold-standard PSG in a variety of objective sleep measures, including in measurements of apnea-hypopnea index (AHI). We demonstrated that AHI, the primary endpoint of this study, was similar between the two systems with high levels of agreement in absolute measurements and relative AHI severity (Cohen’s kappa = 0.97). Agreement between the two systems was also seen in secondary parameters and across several statistical techniques, including Pearson’s correlation, Bland-Altman analyses, and Passing-Bablok Regression (Fig. 2; Tables 2 and 4). It should be noted that while our predefined criteria of a deviation of < 15% is generally clinically acceptable, smaller differences near diagnostic thresholds may lead to diagnostically significant differences. However, when evaluating the similarity of severity levels between the Nox and Vlab devices, the two devices were largely in agreement, despite the inherent complexities of the signals and the role of technicians in manually marking events.

The population used in this study was enrolled following clinician referral to the sleep lab, and therefore this study was performed in the target patient group who typically undergo full sleep study testing. Moreover, our demographic analyses demonstrated that the patients included in our analysis had a diverse set of comorbidities and were not found to have systematic differences according to these comorbidities in our primary endpoint of AHI. Therefore, our findings are likely to be generalizable to the broader population. Overall, the device exceeded our predefined, stringent acceptability thresholds during our trials. Importantly, no adverse events occurred while using the device.

Both devices were consistent in classifying key parameters such as AHI and ODI, in recording total sleep time and efficiency, and in more complex scoring of sleep stages. However, we did note significant differences in REM latency, where the Vlab device indicated a longer time for participants to enter the first REM phase compared to the Nox A1 device.

REM latency differences can be attributed to several key effects due to scoring challenges [20], such as short initial REM periods, inconsistent scoring criteria, subtle eye movements during sleep, and complications in identifying transitions from NREM to REM sleep. Longer REM latencies may have clinical implications for diagnoses of sleep disorders such as narcolepsy and REM sleep behavior disorders and may ultimately lead to missed or delayed diagnoses of these disorders. To better evaluate our data, we performed further analyses, which revealed four outlying data points in REM sleep latency values. This analysis revealed that differences in REM latency (calculated based on absolute time of first REM sleep and first period of sleep) were largely driven not by identification of REM episodes (absolute time of REM sleep had an average difference of 2 min between Vlab and Nox devices), but by inconsistent scoring of sleep latency times. These inconsistencies led to large differences and ultimately to large differences in REM latency measurements in these patients and to significant differences (*p* < 0.05) in the population tested. Additional data collection should be done to determine the reliability of REM sleep latency using the DormoTech Vlab device, especially in specific phenotypic groups whose diagnoses depend on this measurement, such as those with REM-predominant OSA, narcolepsy, or in women. Moreover, repeated scoring of the same dataset by the same or different scorers may help to eliminate scoring errors and potentially reduce these discrepancies.

### Limitations of the study

We recognize several limitations in this study that should be considered. The number of participants in the study used to compare the two methods, while adequate in size to compare overall differences in results from the Nox and Vlab methods, is modest and therefore limits the ability to conduct strong subgroup analyses and generalize our findings to populations with comorbidities that may not have been accounted for here. Moreover, as this clinical trial was a single-country, multi-center study, the findings may need to be validated in other populations. This study was restricted to adult participants (over 22 years old), and therefore further research should be done to determine the usability of the Vlab device for younger patients. Additionally, this study included only single-night in-lab recordings without repeat measurements or consecutive-night testing. Future studies with the Vlab device will need to be performed in the patient’s normal sleep environment (i.e., at home) to assess the usability of the device outside of the sleep laboratory. While our results indicate high similarity in most measurements, REM latency was significantly different between the two populations, largely due to the subjective nature of manual sleep scoring. Finally, an important consideration of this study is that wearing both the Nox and Vlab devices simultaneously may have led to alterations in participants’ sleep architecture and impacted the absolute values of the results. However, this study limitation would not have an impact on the similarities of results in individual participants between the Nox and Vlab devices and does not affect the overall conclusions about the reliability of the Vlab device in measuring sleep signals.

## Conclusion

In a cohort of adult participants referred for full PSG, we demonstrated that the DormoTech Vlab portable sleep data recorder yields clinically near-identical sleep study results to formal, gold-standard PSG recordings. Moreover, via subject-completed questionnaires, we demonstrated that the device is easy to set up, use, and is comfortable during sleep. While future studies will need to be completed to evaluate the use of the Vlab portable device in the home environment, the findings from this clinical trial suggests that the new FDA-cleared Vlab device may open the door to comprehensive, comfortable, and self-applied sleep studies to provide clinicians with accurate, comprehensive, and objective sleep studies for diagnosis and monitoring of treatments.

## Electronic supplementary material

Below is the link to the electronic supplementary material.

Supplementary Material 1

Supplementary Material 2

## Data Availability

The data recorded and analyzed in this study cannot be freely provided due to participant privacy in accordance with ethical guidelines and written informed consent provisions.
