# Supplementary material for: Validation of a novel mask-based device for monitoring of comprehensive sleep parameters and sleep disordered breathing
Source: Sleep Breath. 2025 Jan 20;29(1):83. doi: 10.1007/s11325-025-03250-1 (PMC11753363; doi:10.1007/s11325-025-03250-1)
Supplement: Supplementary file 1 — Supplementary Material 1 [file 11325_2025_3250_MOESM1_ESM.pdf]

Supplementary 1

Title: Validation of a Novel Mask-based Device for Monitoring of Comprehensive Sleep Parameters and Sleep Disordered Breathing

Journal: Sleep and Breathing

Author Information: Benjamin D. Fox, MD[1,3], Murad Shihab, MD[1] Abed Nassir[2], Ofer Barnea, Ph.D[3], Asher Tal, MD[4]

[1]Shamir Medical Center-Be'er Ya'akov, Israel [2]Dormotech Medical,Dolev 4, Raanana, Israel [3]Tel Aviv University Chaim Levanon St 55, Tel Aviv-Yafo, Israel, [4] Soroka Medical Center, Yitzhack I. Rager Blvd. 151, Be'er Sheva, Israel

Corresponding Author: Prof. Asher Tal, Astal2001@gmail.com, *Ben-Gurion University of the Negev, David Ben Gurion Blvd 1, Beer-Sheva, Israel, Tel +972524600554*

Supplementary 1: Patient comorbidities as reported before full polysomnography. Presented as number of participants reporting and percent of all participants initially enrolled in study.

**Patient comorbidities of all enrolled participants (n = 47)**		
None (count, %)	22	46.8%
Liver dysfunction (count, %)	0	0%
Renal dysfunction (count, %)	0	0%
Anemia (count, %)	0	0%
Diabetes (count, %)	6	12.8%
PVD (count, %)	0	0%
COPD (count, %)	0	0%
Neurological (count, %)	0	0%
OSA (AHI > 15) (count, %)	2	4.3%
Hypertension (count, %)	7	14.9%
Other (incl. coronary artery bypass surgery, asthma, osteoporosis, gout, esophageal reflux, hemithyroidectomy, obesity, hyperuricemia, chronic pain, ulcer, migraines, acid reflux, vertigo, hyperthyroidism)	10	21.3%
Did not report	5	10.6%
