# Supplementary material for: Validation of a novel mask-based device for monitoring of comprehensive sleep parameters and sleep disordered breathing
Source: Sleep Breath. 2025 Jan 20;29(1):83. doi: 10.1007/s11325-025-03250-1 (PMC11753363; doi:10.1007/s11325-025-03250-1)
Supplement: Supplementary file 2 — Supplementary Material 2 [file 11325_2025_3250_MOESM2_ESM.pdf]

Supplementary 2

Title: Validation of a Novel Mask-based Device for Monitoring of Comprehensive Sleep Parameters and Sleep Disordered Breathing

Journal: Sleep and Breathing

Author Information: Benjamin D. Fox, MD[1,3], Murad Shihab, MD[1] Abed Nassir[2], Ofer Barnea, Ph.D[3], Asher Tal, MD[4]

[1]Shamir Medical Center-Be'er Ya'akov, Israel [2]Dormotech Medical,Dolev 4, Raanana, Israel [3]Tel Aviv University Chaim Levanon St 55, Tel Aviv-Yafo, Israel, [4] Soroka Medical Center, Yitzhack I. Rager Blvd. 151, Be'er Sheva, Israel

Corresponding Author: Prof. Asher Tal, Astal2001@gmail.com, Ben-Gurion University of the Negev, David Ben Gurion Blvd 1, Beer-Sheva, Israel, Tel +972524600554

Supplementary 2 – Full length questionnaires given to participants to complete before (night) and after (morning) full night of recording. Participants were asked to answer only about their experience with the Vlab device.

Questionnaire - Night

<u>Rate your agreement with the following statements and add details if needed.</u>

1. The head unit is comfortably positioned on the head.

Strongly disagree	Disagree	neutral	Agree	Strongly agree
1	2	3	4	5

2. The head unit is stably positioned on the head.

Strongly disagree	Disagree	neutral	Agree	Strongly agree
1	2	3	4	5

3. Using the head unit does not cause any discomfort.

Strongly disagree	Disagree	neutral	Agree	Strongly agree
1	2	3	4	5

4. No adaptation time is needed for the head unit to rest comfortably.

Strongly disagree	Disagree	neutral	Agree	Strongly agree
1	2	3	4	5

5. Wearing the head unit isn't causing any nasal congestion for me.

Strongly disagree 1	Disagree 2	neutral 3	Agree 4	Strongly agree 5

6. The body unit is comfortably positioned on the chest and abdomen.

Strongly disagree 1	Disagree 2	neutral 3	Agree 4	Strongly agree 5

7. The body unit is stably positioned on the chest and abdomen.

Strongly disagree 1	Disagree 2	neutral 3	Agree 4	Strongly agree 5

8. Using the body unit isn't causing any discomfort/pressure on the body.

Strongly disagree 1	Disagree 2	neutral 3	Agree 4	Strongly agree 5

9. No adaptation time is needed for the body unit to rest comfortably.

Strongly disagree 1	Disagree 2	neutral 3	Agree 4	Strongly agree 5

10. The investigator's instructions were clear to me.

Strongly disagree 1	Disagree 2	neutral 3	Agree 4	Strongly agree 5

11. I had an easy time following the instructions.

Strongly disagree 1	Disagree 2	neutral 3	Agree 4	Strongly agree 5

12. The user Manual was clear.

Strongly disagree 1	Disagree 2	neutral 3	Agree 4	Strongly agree 5

13. The operation of the device was easy and clear.

Strongly disagree 1	Disagree 2	neutral 3	Agree 4	Strongly agree 5

14. The process of establishing the device's connectivity was easy and clear.

Strongly disagree 1	Disagree 2	neutral 3	Agree 4	Strongly agree 5

15. I was able to use the device without requiring assistance.

Strongly disagree	Disagree	neutral	Agree	Strongly agree
1	2	3	4	5

16. What rating would you assign to the device?

Strongly disagree	Disagree	neutral	Agree	Strongly agree
1	2	3	4	5

Questionnaire-Morning

Rate your agreement with the following statements and add details if needed.

1. The head unit was comfortably positioned on the head during the night.

Strongly disagree	Disagree	neutral	Agree	Strongly agree
1	2	3	4	5

2. The head unit was stably positioned on the head during the night.

Strongly disagree	Disagree	neutral	Agree	Strongly agree
1	2	3	4	5

3. Using the head unit didn’t cause any discomfort.

Strongly disagree	Disagree	neutral	Agree	Strongly agree
1	2	3	4	5

4. No adaptation time was needed for the head unit to rest comfortably.

Strongly disagree	Disagree	neutral	Agree	Strongly agree
1	2	3	4	5

5. Wearing the head unit didn’t cause any nasal congestion for me.

Strongly disagree	Disagree	neutral	Agree	Strongly agree
1	2	3	4	5

6. The body unit was comfortably positioned on the chest and abdomen during the night.

Strongly disagree	Disagree	neutral	Agree	Strongly agree
1	2	3	4	5

7. The body unit was stably positioned on the chest and abdomen during the night.

Strongly disagree	Disagree	neutral	Agree	Strongly agree
1	2	3	4	5

8. Using the body unit didn’t cause any discomfort/pressure on the body.

Strongly disagree	Disagree	neutral	Agree	Strongly agree
1	2	3	4	5

9. No adaptation time was needed for the body unit to rest comfortably.

Strongly disagree	Disagree	neutral	Agree	Strongly agree
1	2	3	4	5

10. The investigator’s instructions were clear to me.

Strongly disagree	Disagree	neutral	Agree	Strongly agree
1	2	3	4	5

11. The instructions were easy to follow, and I'm confident doing it on my own.

Strongly disagree	Disagree	neutral	Agree	Strongly agree
1	2	3	4	5

12. The user Manual was clear.

Strongly disagree	Disagree	neutral	Agree	Strongly agree
1	2	3	4	5

13. The operation of the device was easy and clear.

Strongly disagree	Disagree	neutral	Agree	Strongly agree
1	2	3	4	5

14. The process of establishing the device's connectivity was easy and clear.

Strongly disagree	Disagree	neutral	Agree	Strongly agree
1	2	3	4	5

15. I was able to use the device without requiring assistance.

Strongly disagree	Disagree	neutral	Agree	Strongly agree
1	2	3	4	5

16. The device remained in place throughout the entire night.

Strongly disagree	Disagree	neutral	Agree	Strongly agree
1	2	3	4	5

17. The device didn't interrupt my sleep.

Strongly disagree	Disagree	neutral	Agree	Strongly agree
1	2	3	4	5

18. What rating would you assign to the device.

Strongly disagree	Disagree	neutral	Agree	Strongly agree
1	2	3	4	5
